# Interpregnancy Interval and Child Health Outcomes in India: Evidence from Three Recent Rounds of National Family Health Survey

**DOI:** 10.1007/s10995-022-03559-3

**Published:** 2022-11-09

**Authors:** Ajit Kumar Kannaujiya, Kaushalendra Kumar, Lotus McDougal, Ashish Kumar Upadhyay, Anita Raj, K S James, Abhishek Singh

**Affiliations:** 1 International Institute for Population Sciences, Mumbai, India; 2 Department of Public Health & Mortality Studies, International Institute for Population Sciences, Mumbai, India; 3 Center on Gender Equity and Health, University of California, San Diego, USA; 4 GENDER Project, International Institute for Population Sciences, Mumbai, India

**Keywords:** interpregnancy interval, neonatal mortality, postneonatal mortality, diarrhea and/or acute respiratory infections, stunting, underweight, mother fixed-effects, India

## Abstract

**Introduction:**

Short interpregnancy interval (IPI) is a well-known risk factor for preterm births and low birth weights. However, research on the association between interpregnancy interval (IPI) and health outcomes in children under age 5 is limited in India. We examined the associations between IPI and five child health outcomes in India.

**Methods:**

We used nationally representative cross-sectional data from three rounds of National Family Health Survey (NFHS) conducted in India during 2005-06, 2015-16 and 2019-21 to examine the associations between IPI [categorized as < 12 months, 12–17 months, 18–23 months (ref), 24–35 months, and 36–59 months] and five child health outcomes – neonatal mortality, postneonatal mortality, diarrhea and/or acute respiratory infections (ARI), stunting, and underweight, for the total sample and, secondarily, using sex-stratified analyses. We used multivariable and mother fixed-effects binary logistic regressions to examine the associations.

**Results:**

3% and 2% of infants died during the neonatal and postneonatal period, respectively. Thirteen, 40, and 37% of children had diarrhea and/or ARI, were stunted, and were underweight, respectively. IPI < 12 months was associated with higher odds of diarrhea and/or ARI (OR: 1.11; 95% CI: 1.05–1.18), stunting (OR: 1.13; 95% CI: 1.08–1.18) and underweight (OR: 1.06; 95% CI: 1.01–1.11). Mother fixed-effects adjustments confirmed these associations and also found that births with IPI of 12–17 months and 36–59 months had higher odds of stunting, and IPI of 12–17 months was also associated with higher odds of underweight.

**Discussion:**

Our findings indicate that IPIs shorter than 12 months are a risk factor for diarrhea and/or ARI, and IPIs shorter than 12 months and 12–17 months are risk factors for stunting and underweight among children under 5 in India. Mother fixed-effects models allowed us to adjust our estimates for unobserved heterogeneity; this has rarely been done before. Increases in birth spacing may improve child health outcomes in India.

## Significance

### What is already known on this subject?

The association of IPI with child health outcomes is not well understood in India. Previous studies on associations of birth interval with child health outcomes have inadequately accounted for unobserved heterogeneity.

### What this study adds?

IPIs shorter than 12 months and 12–17 months were associated with higher odds of stunting and underweight. While IPIs of 12–17 months and 36–59 months had higher odds of stunting, IPIs of 12–17 months had higher odds of underweight. Our study extends the literature by examining how different IPIs relate to child health outcomes in the first five years of life in a large and diverse country like India.

## Introduction

Mortality, morbidity, and malnutrition among children under age 5 years are key challenges facing India. India contributes considerably to the global numbers of infant mortality, prevalence of common childhood morbidities, and child malnutrition. Recent estimates suggest that India alone accounted for 20% of global neonatal and 17% of global infant deaths. Moreover, the Indian infant mortality rate (IMR), at 32 infant deaths per 1,000 live births, is the highest of neighboring countries such as Nepal, Bangladesh, and Sri Lanka (ORGCCI, 2020; UNIGME, 2017). India also suffers from considerable burden of diarrheal diseases and acute respiratory infections (ARI). In 2016, India alone accounted for 19% of the global deaths from pneumonia and diarrhea among children under age 5 (IVAC & JHBSPH, 2018). In terms of numbers, 0.26 million children under age 5 died of pneumonia (0.16 million) and diarrhea (0.10 million) in 2016 (IVAC & JHBSPH, 2018), comprising 20% of total under-5 deaths in India (IVAC & JHBSPH, 2018). India is also home to 46.6 million stunted children, accounting for 31% of the global stunting burden among children (Development Initiatives 2018). India also has a considerable burden of underweight and wasted children. According to the most recent National Family Health Survey 2019-21 (NFHS-5), 32% and 19% of children under age 5 were underweight and wasted, respectively (IIPS & ICF, 2017, 2022).

Birth interval is a key factor associated with mortality, morbidity, and malnutrition among children under age 5. Birth intervals of < 18 months, < 24 months, and 18–35 months have all been associated with an elevated risk of early neonatal, neonatal, postneonatal, infant, and under 5 mortality in India (Arulampalam & Bhalotra, 2006; Kumar et al., 2013; Molitoris et al., 2019; Rutstein, 2005; van der Klaauw & Wang, 2011; Whitworth & Stephenson, 2002; Williams et al., 2008). Multiple studies in the Indian context have also reported association between short birth intervals and higher risk of stunting and underweight among children under age 5 (Chungkham et al., 2020; Dhingra & Pingali, 2021; Rana et al., 2019; Rana & Goli, 2018; Rutstein, 2005).

While birth interval is a reasonably good indicator of birth spacing, it ignores those pregnancies that result into miscarriage, abortion or stillbirth between two consecutive live births, and is thus not likely the best representation of women’s recuperative potential. In addition, the effects of preterm births may be misattributed to the effects of short birth intervals (Molitoris et al., 2019). Interpregnancy interval (IPI), defined as the duration from the outcome of a given pregnancy to the conception of the subsequent pregnancy, is better able to overcome these limitations associated with birth interval. Several studies from developed and a few developing countries have examined associations between IPI and adverse child health outcomes (Adams et al., 1997; Barclay et al., 2020; Conde-Agudelo et al., 2005; DaVanzo et al., 2007; Klebanoff, 2017; Swaminathan et al., 2020; Zhu & Le, 2003). However, only three studies from India examined associations between IPI and low birth weight (Kader & Perera, 2014; Kannaujiya et al., 2020; Mavalankar et al., 1992) and only one examined the association between IPI and stillbirth (Swaminathan et al., 2020). We could not identify any study from India that has examined the association of IPI with mortality and malnutrition in children under age 5. Although a few Indian studies have examined the association of birth interval with mortality and malnutrition outcomes, questions remain whether previous studies have adequately accounted for unobserved heterogeneity. In addition, a number of these studies are based on data that are dated or are not nationally representative. Finally, despite prior research from India documenting effects of child sex on both birth spacing and child health outcomes in India (Chalasani & Rutstein, 2014; Edmeades et al., 2012; Raj et al., 2015, 2019; Rutstein, 2005; Vilms et al., 2017), with girls being more vulnerable across issues, little analysis on the relationship between IPI and these child health outcomes has been conducted.

Based on these gaps in the science, our study examined the associations of IPI with five child health outcomes – neonatal and postneonatal mortality, diarrhea and/or acute respiratory infections (ARI), stunting, and underweight – in India using the third round of NFHS conducted in 2005-06 (NFHS-3), the fourth round of NFHS conducted in 2015-16 (NFHS-4), and the fifth round of NFHS conducted in 2019-21 (NFHS-5). Our inclusion of diarrhea and/or ARI, stunting, and underweight allowed us to examine the effect of IPI on child health outcomes beyond the first year of life.

## Data and Methods

### Data

Our study used pooled data from NFHS-3, NFHS-4, and NFHS-5, nationally representative household surveys covering over 99% of India’s population. Interviews in NFHS-3, NFHS-4, and NFHS-5 were conducted with 124,385, 699,686, and 724,115 women age 15–49, with respective response rates of 95%, 97%, and 97% (IIPS & ICF, 2007, 2017, 2022). NFHS provides information on maternal and child health, family planning, reproductive health and sexual behavior, and HIV/AIDS knowledge, attitudes, and behavior. NFHS data were collected via face-to-face interviews conducted by trained, sex-matched interviewers, and informed consent was obtained prior to interview. The International Institute for Population Sciences (IIPS) was the nodal agency for managing and conducting the survey under the stewardship of the Ministry of Health and Family Welfare, Government of India.

NFHS-3, NFHS-4, and NFHS-5 adopted a stratified two-stage sampling design. The urban and rural samples within each state were drawn separately. In each state, the rural sample was selected in two stages, with the selection of Primary Sampling Units (PSUs), which are villages, with probability proportional to population size (PPS) selection at the first stage, followed by random selection of households within each PSU in the second stage. In urban areas, a two-stage procedure was followed. In the first stage, census enumeration blocks (CEB) were randomly selected with PPS. In the second stage, households were randomly selected within each selected CEB.

These three rounds of NFHS used standardized questionnaires. Field supervisors conducted spot-checks to verify the accuracy of key information, particularly with respect to the eligibility of respondents. IIPS also appointed one or more research officers in each state for monitoring and supervision throughout the training and fieldwork period to ensure adherence to survey procedures and protocols, and to maintain data quality. Further details regarding sampling design, survey instruments, and field procedures are available in the national NFHS reports (IIPS & ICF, 2007, 2017, 2022). NFHS surveys use uniform sampling design, survey instruments, and field procedures, which allowed us to pool data from the three rounds.

### Ethics and Data Availability Statement

NFHS data are deidentified prior to sharing, and are publicly available at https://dhsprogram.com/. Ethical exemption for this analysis of publicly available, deidentified data was provided by the Institutional Review Board of the University of California San Diego.

### Analytical Sample

We used reproductive calendar data collected in the three surveys to estimate IPI. The reproductive calendar includes a monthly history of key events such as births, pregnancies, pregnancy terminations, contraceptive use, and reasons for contraceptive discontinuation for up to 80 months prior to interview. We considered only reproductive histories of five years (up to 59 months) to minimize self-reporting errors.

**Fig. 1:**
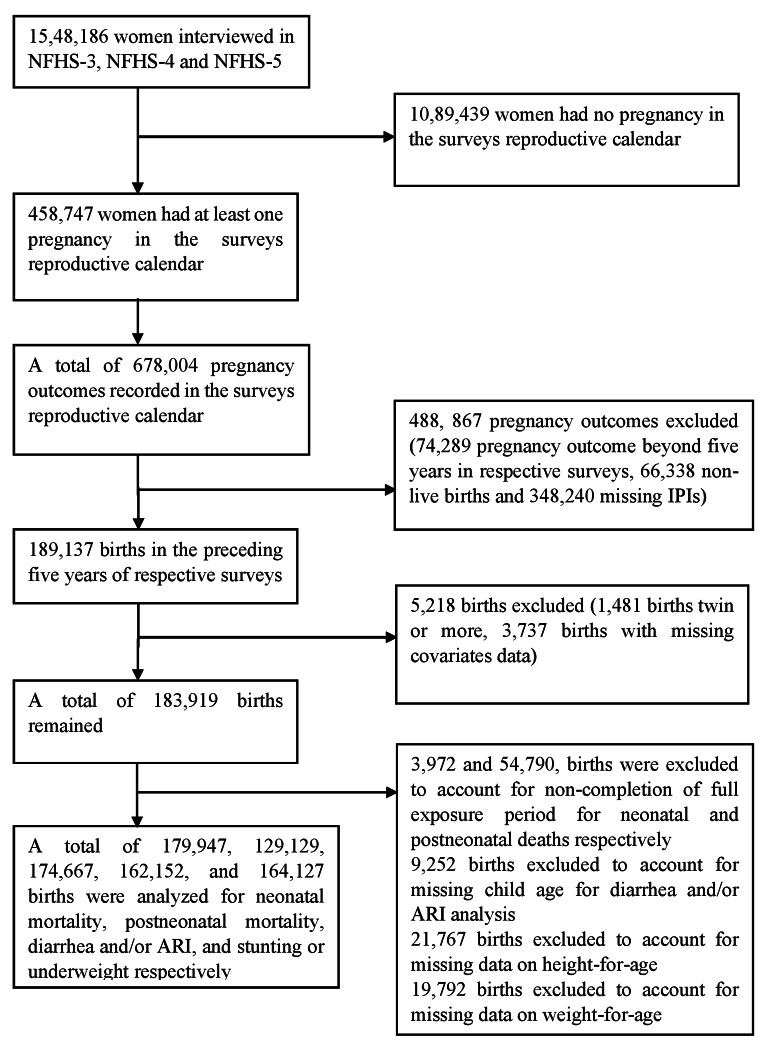
Sample selection process

Our analysis included only those women who had reported at least two pregnancy outcomes in the five years before the survey. Of the total 15,48,186 women interviewed (124, 385, 699,686, and 724,115 women in NFHS-3, NFHS-4, and NFHS-5, respectively), 10,89,439 women reported no pregnancy in the reference period and hence were excluded from the analysis. The remaining women (458,747) contributed a total of 678,004 pregnancies in the reproductive calendar. Of these pregnancies, 488,867 were excluded due to pregnancy outcome beyond the reference period (74,289), all pregnancies of a women in the reference period resulted in a non-live birth (66,338), and women with only one pregnancy in the reference period (348,240). In addition, non-singleton births and births with missing covariates information were also excluded, resulting in an analytic sample size of 183,919 births. The analytic sample for the mortality analyses further excluded 3,972 and 54,790 births that occurred in the past 28 days and in the past 12 months to account for the incomplete exposure time for experiencing neonatal mortality and postneonatal mortality, respectively (n = 179,947 for neonatal mortality and 129,129 for postneonatal mortality). The analytic samples for diarrhea and/or ARI excluded 9,252 births with missing child age, for a sample size of 174,667. The analytic sample for malnutrition outcomes excluded 21,767 and 19,792 births with missing information on height-for-age and weight-for-age z-scores, respectively for a sample size of 162,152 and 164,127 births. Details of the analytical sample are given in Fig. 1.

### Dependent Variables

The dependent variables of the interest were neonatal deaths, postneonatal deaths, occurrence of diarrhea and/or ARI, stunting, and underweight. Neonatal death was coded as ‘1’ if the child died within 28 days of birth, and ‘0’ otherwise. Postneonatal death was coded as ‘1’ if the child died between the 29th day and 1st birthday, and ‘0’ otherwise. Occurrence of diarrhea and/or ARI was coded as ‘1’ if the child suffered any episode of diarrhea and/or ARI in the two weeks prior to interview, and ‘0’ otherwise. Stunting is coded as ‘1’ if the height-for-age z-score is below minus two standard deviations (-2 SD) from the median of the reference population, and ‘0’ otherwise. Likewise, underweight is coded as ‘1’ if the weight-for-age z-score is below minus two standard deviations (-2 SD) from the median of the reference population, and ‘0’ otherwise. Calculation of stunting and underweight are based on the international reference population released by WHO in April 2006 (and accepted by the Government of India (World Health Organization, 2006).

### Independent Variable

IPI, which is defined as the gap between the first month the index pregnancy was reported in the reproductive calendar (referred to as the month of conception) and the month of pregnancy outcome (including live births and terminations) of the preceding pregnancy, is the independent variable. IPI was categorized into 5 groups: <12 months, 12–17 months, 18–23 months, 24–35 months, and 36–59 months.

### Control Variables

Based on existing literature, a number of mother-, child-, household-, and residence- related variables were included in the statistical models. Mother-related variables were mother’s age at conception (< 20 years, 20–24 years, 25–29 years, ≥ 30 years), mother’s height (< 145 centimeters, ≥ 145 centimeters), and mother’s schooling (no schooling, up to primary, higher than primary). Child-related variables were birth order (1, 2, 3, 4, ≥ 5), sex of the child (male, female; also included as a stratification variable), and child wanted (no, yes). Household-related variables included wealth quintiles (poorest, poorer, middle, richer, richest), religion (Hindu, Muslim, others), and caste (scheduled castes, scheduled tribes, other backward classes, others). Residence-related variables included urban-rural residence (urban, rural) and state-region (north, central, east, northeast, west, south). We also controlled for round of the survey (3, 4 and 5).

Child wantedness was constructed using the following two questions canvassed in NFHS-3, NFHS-4, and NFHS-5: *When you got pregnant with (Name), did you want to get pregnant at that time?* (Yes, No). If the women answered no, the follow-up question was: *Did you want to have a baby later on, or did you not want any (more) children?* (Later, No more)

Births for whom the mothers answered ‘no more’ were coded as ‘no’; all remaining births were coded as ‘yes’. Household wealth quintiles are provided in the respective NFHS datasets. The detail of the state-region variable is given in Appendix Table A1.

### Methods

Since all five dependent variables are binary, we estimated multivariable binary logistic regressions to examine the associations. We further used mother fixed-effects multivariable binary logistic regressions to account for the unobserved heterogeneity. In the mother fixed-effects regressions, we included only those sibling pairs in which one sibling had an outcome different from that of the other sibling. Finally, we used sex-stratified multivariable binary logistic regression models to assess the associations between IPI and five child health outcomes.

We adjusted all estimates for the complex survey design used in NFHS-3, NFHS-4, and NFHS-5, and used appropriate weights in the estimations. We carried out all the estimations in Stata 16.1.

## Results

### Descriptive Results

Descriptive statistics of births occurring in the five years prior to interview for each of the five outcome variables are shown in Table 1. Between 34 and 38%, 25–27%, 17%, 15–18%, and 3–7% of the births had IPIs of < 12 months, 12–17 months, 18–23 months, 24–35 months, and 36–59 months, respectively. The percent distribution of all control variables was similar across the five samples. About 7% of births were first order births. About half (48–50%) of the births were second order births and about 13% were reported unwanted by their mothers. About 23% of births were from urban areas. Between 42 and 44%, 43–45%, and 12–15% of births in the pooled sample came from NFHS-5, NFHS-4, and NFHS-3 respectively. Distribution of male and female births by IPI were similar (Appendix Table A2).

**Table 1:** Interpregnancy interval and background characteristics of children born in past five years, India, NFHS 2019-21, NFHS 2015-16, and NFHS 2005-06 (pooled data)

Covariate & category	Neonatal Mortality sample (1,79,947)		Postneonatal Mortality sample (1,29,129)		Diarrhea and/or ARI sample (1,74,667)		Stunting sample (1,62,152)		Underweight sample (1,64,127)	
	**Percent**	**N**	**Percent**	**N**	**Percent**	**N**	**Percent**	**N**	**Percent**	**N**
**Interpregnancy interval**										
< 12 months	34.4	59,929	38.2	47,947	33.5	56,646	33.7	53,021	33.6	53,492
12–17 months	25.4	45,801	27.0	35,153	25.4	44,444	25.6	41,592	25.5	41,980
18–23 months	16.8	30,786	16.7	22,099	17.0	30,160	17.0	28,012	17.0	28,348
24–35 months	17.2	31,505	15.1	19,827	17.5	31,199	17.4	28,676	17.5	29,159
36–59 months	6.3	11,926	3.1	4,103	6.6	12,218	6.4	10,851	6.5	11,148
**Mother’s age at conception**										
< 20 years	8.2	12,424	9.2	10,047	8.0	11,776	8.0	10,903	8.0	10,999
20–24 years	47.3	81,043	48.1	59,561	47.3	78,690	47.6	73,430	47.5	74,225
25–29 years	31.3	58,474	30.0	40,323	31.6	57,106	31.5	52,911	31.6	53,641
≥ 30 years	13.2	28,006	12.7	19,198	13.1	27,095	12.9	24,908	13.0	25,262
**Mother’s height**										
< 145cm	13.3	22,951	13.3	16,443	13.1	21,976	13.1	20,361	13.1	20,655
≥ 145cm	86.7	1,56,996	86.7	1,12,686	86.9	1,52,691	86.9	1,41,791	86.9	1,43,472
**Mother’s education**										
No schooling	34.4	60,158	36.4	45,144	33.8	57,437	33.5	52,828	33.5	53,351
Primary	14.6	27,585	14.8	20,020	14.6	26,644	14.6	24,781	14.6	25,052
Secondary or Higher	50.9	92,204	48.8	63,965	51.6	90,586	51.8	84,543	51.9	85,724
**Birth order**										
1	6.6	11,115	6.8	8,141	6.6	10,764	6.7	10,127	6.7	10,233
2	48.9	86,061	48.1	60,980	49.4	84,213	49.4	78,339	49.5	79,340
3	22.9	41,783	22.7	29,772	22.8	40,577	22.8	37,648	22.8	38,119
4	10.8	20,388	11.0	14,797	10.8	19,634	10.8	18,174	10.8	18,402
≥ 5	10.7	20,600	11.4	15,439	10.4	19,479	10.3	17,864	10.2	18,033
**Sex of the child**										
Male	52.0	93,600	52.0	67,241	52.0	90,815	51.8	83,958	51.8	85,060
Female	48.0	86,347	48.0	61,888	48.0	83,852	48.2	78,194	48.2	79,067
**Child wanted**										
No	13.2	22,278	13.2	15,926	13.0	21,351	13.0	19,714	12.9	19,865
Yes	86.8	1,57,669	86.8	1,13,203	87.0	1,53,316	87.0	1,42,438	87.1	1,44,262
**Wealth quintiles**										
Poorest	29.5	54,425	30.1	39,702	29.1	52,386	29.1	48,506	29.1	49,145
Poorer	23.7	44,222	23.9	32,001	23.6	42,770	23.7	39,758	23.7	40,234
Middle	19.7	35,020	19.6	24,997	19.7	34,099	19.9	31,766	19.9	32,133
Richer	16.3	27,623	16.1	19,573	16.5	27,005	16.5	25,149	16.5	25,431
Richest	10.9	18,657	10.3	12,856	11.1	18,407	10.9	16,973	10.9	17,184
**Religion**										
Hindu	78.3	1,29,024	78.2	92,431	78.2	1,25,154	78.4	1,16,594	78.4	1,18,032
Muslim	17.6	29,197	17.9	21,335	17.6	28,289	17.5	26,127	17.5	26,457
Others	4.1	21,726	4.0	15,363	4.1	21,224	4.1	19,431	4.1	19,638
**Caste**										
Scheduled Caste	23.5	37,093	23.6	26,669	23.5	35,829	23.6	33,356	23.6	33,774
Scheduled Tribe	10.5	36,543	10.4	25,939	10.5	35,644	10.4	32,670	10.4	33,095
Other Backward Class	44.5	70,021	44.6	50,400	44.5	67,899	44.6	63,423	44.6	64,194
Others	21.5	36,290	21.4	26,121	21.5	35,295	21.5	32,703	21.4	33,064
**Place of residence**										
Urban	23.2	36,804	23.1	26,370	23.4	35,871	23.1	32,982	23.1	33,359
Rural	76.8	1,43,143	76.9	1,02,759	76.6	1,38,796	76.9	1,29,170	76.9	1,30,768
**Region**										
North	12.8	31,696	12.6	22,565	12.8	30,835	12.8	28,680	12.8	29,012
Central	31.2	54,172	31.7	39,291	30.7	51,956	31.0	48,734	31.0	49,273
East	26.3	37,442	26.3	26,895	26.3	36,361	26.7	34,158	26.7	34,542
Northeast	3.0	24,508	3.0	17,426	3.0	23,964	3.0	21,935	3.0	22,187
West	11.5	13,596	11.3	9,764	11.6	13,382	11.4	12,134	11.4	12,318
South	15.4	18,533	15.2	13,188	15.6	18,169	15.2	16,511	15.2	16,795
**Survey round**										
Third	13.0	19,975	14.7	15,739	12.8	19,056	12.3	16,845	12.1	16,845
Fourth	44.5	83,663	43.2	58,527	44.5	81,021	44.2	74,708	43.7	74,708
Fifth	42.4	76,309	42.1	54,863	42.8	74,590	43.6	70,599	44.2	72,574

**Fig. 2:**
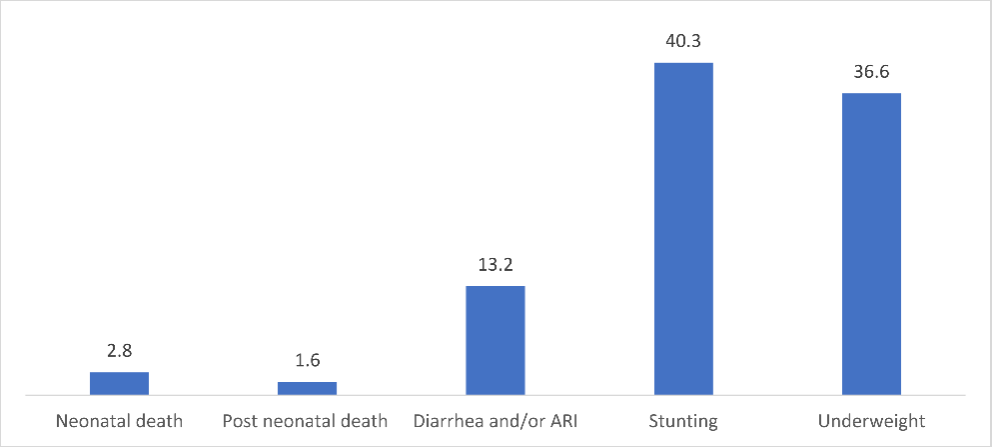
Child health outcomes for the children born in past five years, India, NFHS 2019-21, NFHS 2015-16, and NFHS 2005-06 (pooled data)

Three percent and two percent of infants died during the neonatal and postneonatal period, respectively. Thirteen, 40, and 37% of children had diarrhea and/or ARI, stunting, and underweight respectively (Fig. 2).

Table 2 shows the distribution of outcomes by IPI. Over 4% of infants with IPIs < 12 months died within 28 days of birth. In comparison, only 2% of infants with IPIs 18–23 months died within 28 days of birth. While 2% of infants with an IPI < 12 months died during the postneonatal period, only 1% infants with IPIs of 18–23 months died during the same period. Importantly, 2% of infants with IPIs 36–59 months died during the postneonatal period. The prevalence of recent diarrhea ranged between 13% among children with an IPI < 12 months and 14% among children with an IPI 36–59 months. Stunting and underweight monotonically decreased with an increase in IPI. 43% and 38% of children born with an IPI < 12 months were respectively stunted and underweight. In comparison, only 28% and 27% of children born with an IPI 36–59 months were stunted and underweight, respectively. The distributions of outcomes by IPI in the male and female samples are shown in Appendix Table A3.

**Table 2:** Distribution of outcomes by interpregnancy interval, socio-economic, and demographic characteristics, India, NFHS 2019-21, NFHS 2015-16, and NFHS 2005-06 (pooled data)

Covariate & category	Neonatal death (1,79,947)	Postneonatal death (1,29,129)	Diarrhea and/or ARI (1,74,667)	Stunting (1,62,152)	Underweight (1,64,127)
**Interpregnancy interval**	***Percent***	***Percent***	***Percent***	***Percent***	***Percent***
< 12 months	3.8	2.0	13.0	42.6	37.7
12–17 months	2.5	1.4	13.1	43.4	38.9
18–23 months	2.1	1.4	13.5	39.8	36.9
24–35 months	2.0	1.3	13.4	36.5	34.3
36–59 months	2.4	2.3	13.7	27.5	27.3
**Mother’s age at conception**					
< 20 years	3.5	1.7	14.2	45.8	40.7
20–24 years	2.6	1.4	13.3	40.6	36.3
25–29 years	2.5	1.7	13.1	38.4	35.1
≥ 30 years	3.8	2.1	12.9	40.5	38.5
**Mother’s height**					
< 145cm	3.7	2.1	13.1	56.2	50.8
≥ 145cm	2.7	1.6	13.2	37.9	34.4
**Mother’s schooling**					
No schooling	3.8	2.3	13.9	49.4	46.2
Up to primary	3.2	1.8	14.5	43.1	38.5
Higher than primary	2.0	1.1	12.5	33.6	29.8
**Birth order**					
1	2.7	0.9	14.2	30.8	27.5
2	2.4	1.2	12.4	37.3	33.4
3	2.8	1.9	13.4	42.2	38.6
4	3.1	2.2	14.7	45.6	42.2
≥ 5	4.7	2.7	14.8	51.0	47.8
**Sex of the child**					
Male	3.0	1.5	13.7	41.2	37.5
Female	2.6	1.8	12.7	39.3	35.6
**Child wanted**					
No	3.6	2.1	18.6	42.6	38.5
Yes	2.7	1.6	12.4	40.0	36.3
**Wealth quintiles**					
Poorest	3.6	2.0	14.2	49.2	47.4
Poorer	3.1	1.9	13.7	43.3	39.3
Middle	2.5	1.5	13.0	38.4	33.8
Richer	2.2	1.2	12.5	32.3	27.3
Richest	1.5	0.9	11.1	25.5	21.1
**Religion**					
Hindu	2.9	1.7	13.0	40.4	37.1
Muslim	2.8	1.6	14.5	41.1	35.7
Other	2.0	1.6	11.1	35.9	30.9
**Caste**					
Scheduled Caste	3.2	1.7	13.4	44.1	39.9
Scheduled Tribe	2.7	1.9	11.6	43.8	44.3
Other Backward Class	2.8	1.6	13.4	39.9	36.1
Other	2.5	1.5	13.6	35.1	30.3
**Place of residence**					
Urban	2.0	1.3	12.2	35.2	30.8
Rural	3.0	1.7	13.5	41.8	38.3
**State-region**					
North	2.6	1.7	12.2	35.1	30.3
Central	3.7	2.3	14.8	43.4	38.4
East	2.9	1.4	14.9	43.4	41.8
Northeast	2.6	1.9	8.7	37.8	30.5
West	1.9	1.2	13.3	40.4	37.9
South	1.6	0.8	9.0	33.4	29.3
**Survey round**					
Third	3.8	2.1	16.2	48.9	43.4
Fourth	2.8	1.6	14.2	40.1	37.4
Fourth	2.5	1.5	11.3	38.1	33.9

### Multivariable Regression Results

Infants with an IPI < 12 months were 1.87 (95% CI: 1.68–2.09) and 1.61 (95% CI: 1.36–1.91) times as likely as infants with an IPI 18–23 months to die during the neonatal and postneonatal periods, respectively (Table 3). Infants with an IPI of 36–59 months were also more likely to die during the postneonatal period 1.73 (95% CI: 1.28–2.33). After controlling for unobserved heterogeneity, infants with an IPI < 12 months were only 0.74 (95% CI: 0.60,0.91) as likely infants with IPIs 18–23 months to die within the first 28 days of birth. Female infants were less likely to die during the neonatal period compared to male infants. Infants reported wanted by their mothers were less likely to die during both windows.

**Table 3:** Logistic regression analysis of association of interpregnancy interval, socio-economic, and demographic characteristics with infant mortality, India, NFHS 2019-21, NFHS 2015-16 and NFHS 2005-06 (pooled data)

Covariate & category	Neonatal death		Postneonatal death	
	**Total sample (1,79,947)**	**Mother fixed-effects** **(Number of groups – 4,629)**	**Total sample (1,29,129)**	**Mother fixed-effects** **(Number of groups – 1,654)**
**Interpregnancy interval**				
< 12 months	1.87*(1.68,2.09)	0.74*(0.60,0.91)	1.61*(1.36,1.91)	0.83(0.59,1.16)
12–17 months	1.16*(1.03,1.31)	0.77*(0.61,0.97)	1.03(0.85,1.25)	0.92(0.63,1.34)
18–23 months®	1.00	1.00	1.00	1.00
24–35 months	0.96(0.83,1.10)	1.07(0.77,1.50)	0.93(0.76,1.14)	0.97(0.54,1.75)
36–59 months	1.20(0.99,1.44)	2.69(0.93,7.75)	1.73*(1.28,2.33)	2.22(0.22,22.61)
**Mother’s age at conception**				
< 20 years	1.25*(1.09,1.43)	0.82(0.60,1.11)	1.30*(1.05,1.60)	1.76*(1.01,3.11)
20–24 years®	1.00	1.00	1.00	1.00
25–29 years	0.92(0.84,1.01)	1.05(0.82,1.35)	1.01(0.86,1.16)	0.96(0.66,1.40)
≥ 30 years	1.14*(1.01,1.31)	0.84(0.52,1.35)	0.98(0.81,1.18)	1.54(0.74,3.22)
**Mother’s height**				
< 145cm	1.25*(1.13,1.38)		1.23*(1.06,1.42)	
≥ 145cm®	1.00		1.00	
**Mother’s schooling**				
No schooling	1.35*(1.22,1.50)		1.52*(1.31,1.77)	
Up to primary	1.29*(1.15,1.46)		1.37*(1.16,1.63)	
Higher than primary®	1.00		1.00	
**Birth order**				
1	0.70*(0.57,0.86)	56.17*(32.72,96.4)	0.40*(0.28,0.56)	1.79(0.75,4.26)
2	0.72*(0.62,0.82)	14.14*(9.65,20.74)	0.60*(0.49,0.74)	2.10*(1.21,3.66)
3	0.76*(0.66,0.87)	4.74*(3.41,6.61)	0.83*(0.69,0.99)	2.49*(1.54,4.03)
4	0.79*(0.68,0.91)	1.68*(1.30,2.17)	0.90(0.74,1.08)	1.51*(1.04,2.20)
≥ 5®	1.00	1.00	1.00	1.00
**Sex of the child**				
Male®	1.00	1.00	1.00	1.00
Female	0.82*(0.77,0.89)	0.64*(0.56,0.73)	1.18*(1.05,1.31)	1.12(0.91,1.37)
**Child wanted**				
No®	1.00	1.00	1.00	1.00
Yes	0.89*(0.81,0.99)	0.32*(0.25,0.40)	0.93(0.80,1.08)	0.56*(0.38,0.82)
**Wealth quintiles**				
Poorest	1.53*(1.23,1.89)		1.49*(1.04,2.12)	
Poorer	1.50*(1.22,1.85)		1.58*(1.12,2.23)	
Middle	1.35*(1.10,1.66)		1.48*(1.05,2.10)	
Richer	1.31*(1.06,1.61)		1.23(0.87,1.76)	
Richest®	1.00		1.00	
**Religion**				
Hindu	1.16(0.94,1.44)		0.84(0.61,1.16)	
Muslim	1.07(0.85,1.35)		0.70*(0.50,0.99)	
Other ®	1.00		1.00	
**Caste**				
Scheduled Caste	1.06(0.94,1.21)		0.90(0.74,1.09)	
Scheduled Tribe	0.88(0.75,1.02)		0.98(0.79,1.22)	
Other Backward Class	0.98(0.87,1.09)		0.96(0.81,1.14)	
Other ®	1.00		1.00	
**Place of residence**				
Urban ®	1.00		1.00	
Rural	1.17*(1.04,1.31)		0.95(0.80,1.13)	
**State-region**				
North	1.49*(1.26,1.76)		1.70*(1.31,2.21)	
Central	1.86*(1.59,2.16)		2.08*(1.63,2.65)	
East	1.39*(1.18,1.63)		1.23(0.94,1.60)	
Northeast	1.40*(1.13,1.74)		1.70*(1.21,2.38)	
West	1.14(0.92,1.40)		1.29(0.91,1.81)	
South ®	1.00		1.00	
**Survey round**				
Third ®	1.00		1.00	
Fourth	0.85*(0.76,0.95)		0.93(0.79,1.10)	
Fifth	0.78*(0.69,0.88)		0.98(0.82,1.17)	

*p < 0.05

Children with an IPI < 12 months were 1.11 (95% CI: 1.05–1.18) times as likely as children born with an IPI 18–23 months to have diarrhea and/or ARI (Table 4). The higher odds of diarrhea and/or ARI of children with IPIs < 12 months increased from 1.11 to 1.25 in the fixed-effects regression. IPIs shorter than 12 months and 12–17 months were associated with higher odds of stunting and underweight in both models. While an IPI of 36–59 months was not associated with higher odds of stunting in the basic regression, this IPI was associated with higher odds of stunting in the fixed-effects regression. Importantly, the higher odds of stunting and underweight among children with IPIs < 12 months increased from basic regression to the fixed-effects regression. Children born with an IPI < 12 months were 1.56 (95% CI: 1.37–1.77) and 1.59 (95% CI: 1.41–1.79) times as likely as children born with an IPI 18–23 months to be stunted and underweight respectively. Likewise, children born with an IPI of 12–17 months were 1.29 (95% CI: 1.14–1.45) and 1.39 (95% CI: 1.24–1.55) times as likely as children born with an IPI 18–23 months to be stunted and underweight, respectively. While children born with an IPI of 36–59 months were more likely to be stunted compared with children born with an IPI of 18–23 months in the fixed-effects regression, children born with an IPI of 24–35 months were less likely to be underweight compared with children born with an IPI of 18–23 months.

**Table 4:** Logistic regression analysis of association of interpregnancy interval, socio-economic, and demographic characteristics with child health outcomes, India, NFHS 2019-21, NFHS 2015-16, and NFHS 2005-06 (pooled data)

Covariate & category	Occurrence of diarrhea and/or acute respiratory infection		Stunting		Underweight	
	**Total sample (1,74,667)**	**Mother fixed-effects** **(Number of groups – 6,898)**	**Total** **sample (1,62,152)**	**(Mother fixed-effects** **(Number of groups – 16,360)**	**Total** **Sample (1,64,127)**	**Mother fixed-effects (Number of groups – 13,890)**
**Interpregnancy interval**						
< 12 months	1.11*(1.05,1.18)	1.25*(1.04,1.49)	1.13*(1.08,1.18)	1.56*(1.37,1.77)	1.06*(1.01,1.11)	1.59*(1.41,1.79)
12–17 months	1.01(0.96,1.07)	0.99(0.83,1.18)	1.08*(1.04,1.13)	1.29*(1.14,1.45)	1.03(0.99,1.08)	1.39*(1.24,1.55)
18–23 months®	1.00	1.00	1.00	1.00	1.00	1.00
24–35 months	0.95(0.89,1.01)	0.76*(0.59,0.98)	1.01(0.97,1.07)	1.11(0.93,1.31)	0.98(0.93,1.02)	0.85*(0.73,0.99)
36–59 months	0.99(0.91,1.08)	0.07*(0.03,0.15)	1.01(0.93,1.08)	1.80*(1.12,2.9)	0.9*(0.84,0.96)	1.35(0.87,2.12)
**Mother’s age at conception**						
< 20 years	1.11*(1.03,1.20)	1.04(0.80,1.35)	1.12*(1.06,1.19)	1.12(0.94,1.34)	1.11*(1.04,1.18)	1.26*(1.06,1.50)
20–24 years®	1.00	1.00	1.00	1.00	1.00	1.00
25–29 years	0.95*(0.90,0.99)	1.04(0.85,1.28)	0.90*(0.87,0.93)	0.96(0.84,1.09)	0.93*(0.90,0.97)	0.98(0.86,1.11)
≥ 30 years	0.88*(0.82,0.94)	1.23(0.82,1.86)	0.84*(0.79,0.88)	0.78(0.58,1.04)	0.92*(0.87,0.97)	0.99(0.76,1.30)
**Mother’s height**						
< 145 cm	0.94*(0.89,0.99)		2.01*(1.92,2.10)		1.77*(1.70,1.85)	
≥ 145 cm®	1.00		1.00		1.00	
**Mother’s schooling**						
No schooling	0.95(0.90,1.01)		1.31*(1.26,1.36)		1.29*(1.24,1.34)	
Up to primary	1.07*(1.01,1.13)		1.15*(1.09,1.20)		1.10*(1.05,1.15)	
Higher than primary®	1.00		1.00		1.00	
**Birth order**						
1	1.12*(1.01,1.25)	0.32*(0.14,0.74)	0.55*(0.51,0.60)	0.10*(0.05,0.18)	0.59*(0.54,0.64)	0.15*(0.09,0.27)
2	0.91*(0.84,0.99)	0.52*(0.28,0.99)	0.74*(0.70,0.79)	0.25*(0.16,0.40)	0.76*(0.72,0.80)	0.29*(0.19,0.44)
3	0.95(0.88,1.03)	0.79(0.50,1.26)	0.82*(0.77,0.87)	0.42*(0.30,0.59)	0.83*(0.79,0.88)	0.47*(0.35,0.65)
4	1.03(0.95,1.11)	1.05(0.79,1.39)	0.87*(0.82,0.93)	0.63*(0.51,0.78)	0.88*(0.83,0.93)	0.69*(0.57,0.84)
≥ 5 ®	1.00	1.00	1.00	1.00	1.00	1.00
**Sex of the child**						
Male®						
Female	0.91*(0.88,0.95)	0.97(0.87,1.08)	0.90*(0.88,0.93)	0.89*(0.82,0.95)	0.90*(0.88,0.93)	0.92*(0.86,0.99)
**Age of the child (in months)**	1.03*(1.02,1.03)	1.03*(1.02,1.05)	1.11*(1.11,1.12)	1.17*(1.16,1.19)	1.05*(1.05,1.06)	1.09*(1.08,1.10)
**Age of child square (in months)**	0.99*(0.99,0.99)	0.99*(0.99,0.99)	0.99*(0.99,0.99)	0.99*(0.99,0.99)	0.99*(0.99,0.99)	0.99*(0.99,0.99)
**Child wanted**						
No®	1.00	1.00	1.00	1.00	1.00	1.00
Yes	0.68*(0.64,0.71)	1.01(0.85,1.20)	0.99(0.95,1.03)	0.78*(0.68,0.88)	1.01(0.96,1.05)	0.82*(0.72,0.92)
**Wealth quintiles**						
Poorest	1.19*(1.08,1.31)		2.11*(1.96,2.27)		2.38*(2.21,2.56)	
Poorer	1.16*(1.07,1.27)		1.80*(1.68,1.93)		1.91*(1.77,2.05)	
Middle	1.15*(1.05,1.25)		1.57*(1.47,1.68)		1.62*(1.51,1.73)	
Richer	1.13*(1.04,1.23)		1.26*(1.17,1.35)		1.26*(1.17,1.35)	
Richest®	1.00		1.00		1.00	
**Religion**						
Hindu	0.99(0.89,1.10)		1.02(0.94,1.10)		1.12*(1.02,1.22)	
Muslim	1.12(0.99,1.26)		1.12*(1.03,1.23)		1.15*(1.04,1.26)	
Others ®	1.00		1.00		1.00	
**Caste**						
Scheduled Castes	1.02(0.96,1.10)		1.29*(1.22,1.35)		1.30*(1.23,1.37)	
Scheduled Tribes	0.88*(0.81,0.96)		1.16*(1.09,1.23)		1.41*(1.33,1.50)	
Other Backward Classes	1.02(0.96,1.08)		1.15*(1.10,1.20)		1.19*(1.14,1.24)	
Others ®	1.00		1.00		1.00	
**Place of residence**						
Urban ®	1.00		1.00		1.00	
Rural	1.04(0.97,1.11)		0.96(0.92,1.01)		0.94*(0.89,0.98)	
**State-region**						
North	1.38*(1.27,1.51)		1.04(0.98,1.11)		0.99(0.93,1.05)	
Central	1.68*(1.55,1.81)		1.20*(1.14,1.27)		1.11*(1.05,1.17)	
East	1.64*(1.51,1.79)		1.03(0.97,1.09)		1.12*(1.05,1.18)	
Northeast	0.91(0.81,1.03)		0.92*(0.85,0.99)		0.77*(0.71,0.83)	
West	1.54*(1.40,1.71)		1.31*(1.22,1.41)		1.40*(1.30,1.51)	
South ®	1.00		1.00		1.00	
**Survey round**						
Third ®	1.00		1.00		1.00	
Fourth	0.87*(0.81,0.93)		0.75*(0.72,0.79)		0.84*(0.80,0.88)	
Fifth	0.70*(0.65,0.75)		0.72*(0.68,0.76)		0.74*(0.70,0.78)	
*p < 0.05; ® Reference						

### Sex-stratified Multivariable Regression Results

Among males, births with an IPI < 12 months were 1.66 (95% CI: 1.43–1.92) and 1.65 (95% CI: 1.28–2.13) times as likely as births with an IPI 18–23 months to die during the neonatal and postneonatal periods. Male births with an IPI of 36–59 months were also more likely to die during the postneonatal period 1.69 (95% CI: 1.12–2.55). Likewise, an IPI shorter than 12 months was associated with higher odds of occurrence of diarrhea and/or ARI and stunting among male children. In addition, IPIs of 12–17 months and 36–59 months were associated with higher odds of stunting among the male children. Among females, children with an IPI < 12 months was associated with higher odds of neonatal mortality, occurrence of diarrhea and/or ARI, stunting, and underweight. Importantly, an IPI of 12–17 months was associated with higher odds of neonatal deaths and stunting among the female children. IPIs of 36–59 months were protective against stunting and underweight among the female children (Appendix Tables A4-A5).

## Discussion

After accounting for unobserved heterogeneity, IPIs shorter than 12 months were associated with higher risk of diarrhea and/or ARI compared with IPIs of 18–23 months. The only comparative study to ours examined hospitalizations among Swedish children, and did not find any association between short IPI and probability of hospitalization. The study further found that IPIs of 43 months or longer decreased the risk of hospitalization among Swedish children (Barclay et al., 2020). In line with these findings, IPIs of 36–59 months substantially decreased the risk of diarrhea and/or ARI among Indian children. IPIs shorter than 18 months were also associated with elevated risk of stunting and underweight relative to IPIs of 18–23 months in our study, a finding that is consistent with the previous studies linking short birth intervals with stunting and underweight in India (Chungkham et al., 2020; Dhingra & Pingali, 2021; Rana et al., 2019; Rana & Goli, 2018; Rutstein, 2005). Importantly, IPIs of 36–59 months were also associated with stunting in our study, a finding that has not been seen in any previous Indian study. Such diminishing returns to lengthening of birth spacing beyond 36 months on infant mortality was observed in Molitoris et al., (2019) (Molitoris et al., 2019).

Unlike previous Indian studies that found association of short birth intervals with neonatal and postneonatal mortality (Arulampalam & Bhalotra, 2006; Kumar et al., 2013; Molitoris et al., 2019; Rutstein, 2005; van der Klaauw & Wang, 2011; Whitworth & Stephenson, 2002; Williams et al., 2008), we did not find any association between short IPIs and neonatal and postneonatal mortality in our study. This difference may be attributable to the accounting of unobserved heterogeneity in our study. IPIs shorter than 12 months were indeed associated with higher risk of neonatal and postneonatal mortality in regressions based on all births. However, we were unable to correctly estimate the association of short IPIs with the two mortality outcomes and diarrhea and/or ARI in fixed-effects regressions due to very small samples. For example, there were only 26 births with IPIs of 36–59 months in the neonatal fixed-effects sample. Likewise, there were very few births with IPIs of 36–59 months in the postneonatal and diarrhea and/or ARI fixed-effects samples (4 and 27, respectively).

Associations between IPIs and child health outcomes varied by the sex of the child in multivariable binary logistic regressions, a finding novel to this study. While IPIs shorter than 12 months were associated with higher risk of neonatal and postneonatal mortality among both males and female infants, IPIs of 12–17 months were associated with higher risk of neonatal deaths only among female infants. Moreover, IPIs of 36–59 months were protective against stunting and underweight among female children. However, we could not confirm these associations with mother fixed-effects analyses due to sample size limitations. These data add to the developing body of literature on differential risks for child health outcomes based on sex of the child in India, findings previously observed in analysis of correlates related to child health outcomes, including wealth, sibling sex composition, and birth order (Chalasani & Rutstein, 2014; Edmeades et al., 2012; Raj et al., 2015, 2019; Vilms et al., 2017).

Importantly, 34–38% of the IPIs in our sample were shorter than 12 months and an additional 25–27% were 12–17 months. Given the high prevalence of such short IPIs in India, the overall population health impact of these short IPIs is likely to be large. Our findings, therefore, call for strategies to tackle the huge burden of short IPIs in India. Increasing focus on spacing methods of family planning in India to reduce the serious consequences of short IPIs is an option that merits further consideration. Female sterilization has been, and remains, the dominant method of family planning in India, with long-acting reversible contraceptive use still quite rare (IIPS & ICF, 2017; Singh 2018; Singh et al., 2012). Antenatal screenings and postpartum checkups offer an important opportunity to provide high-quality, patient-centered counseling on family planning methods, and indeed, have been shown to be associated with postpartum contraceptive uptake (McDougal et al., 2020). Interestingly, only 69% of women age 15–49 with a live birth in the five years preceding the NFHS-4 who met with a community health worker in the last three months of pregnancy for their most recent live birth received advice on family planning (IIPS & ICF, 2017). Moreover, significant socio-economic inequalities exist in receipt of advice on family planning (Singh et al., 2012). Taken together, there is ample scope to increase prevalence of contraceptive counselling, and improve the content of that counselling.

A related key finding that deserves discussion is the association between birth wantedness and child health outcomes. Unwanted births were more likely than wanted births to die during the neonatal and postneonatal periods, be stunted or underweight in our pooled analysis. This finding is consistent with previous research (Singh et al., 2012, 2013, 2017), and suggests that unwanted births continue to face higher risks of poor child health outcomes in India. Access to effective family planning can thus play an important role in reducing the burden of unwanted or mistimed births in India.

Our study has limitations, which must be noted. First, we could not estimate the association between IPIs longer than 59 months and the five child health outcomes due to data limitations. Second, the mother fixed-effects analyses come with some cost. The sample size reduces drastically in fixed-effects regressions. In our case, the sample size even dropped further because we included only those sibling pairs in the fixed-effects in which one sibling has an outcome different from that of the other sibling. Moreover, the fixed-effects sample was more likely to be comprised of uneducated, poorer, and urban-residing mothers, all of which were controlled for in our standard regression models. This was particularly true in the case of our mortality analyses. Third, ARI and/or acute diarrhea outcomes are the weakest primary outcomes in our study, as they measure a single occurrence in time rather than cumulative episodes of illness over the child’s life. Finally, as NFHS data are based on interviews, reporting and recall bias in the reproductive calendar are possible on the part of mother. Since no research has systematically examined the accuracy of the reproductive calendar for estimating IPIs, it remains difficult to assess the effect of such biases on our estimates.

This study makes several key contributions. Prior research examining associations between IPI length and health outcomes has been generally limited to the first year of life; we extend this knowledge base by examining these relationships over the course of the first five years of life in a large and diverse country. In addition, we used high-quality, large-scale, representative household survey data and advanced statistical models, such as mother fixed-effects models, to examine these associations. Further, mother fixed-effects models allowed us to adjust our estimates for unobserved heterogeneity, which has rarely been done in previous research. As we were unable to accurately estimate the association between IPI length and mortality during infancy despite very large sample sizes, future research would benefit from more statistically efficient methodologies to examine associations of IPI length with mortality during infancy and early childhood while accounting for potential biases. Such innovative approaches may also be used to confirm the sex-stratified associations of IPI length with the five child health outcomes noted in our study. In addition, future research may also explore the relative importance of IPI length for other important child health outcomes in low- and middle-income countries such as India.

## Data Availability

Indian Demographic and Health Survey (National Family Health Survey: NFHS) data are deidentified prior to sharing, and are made publicly available at https://dhsprogram.com/.

## Appendix

**Table A1:** States included in various categories of state-region

Category	States included
North	Chandigarh, Haryana, Himachal Pradesh, Jammu and Kashmir, Ladakh, Delhi, Rajasthan, Punjab, Uttarakhand
Central	Chhattisgarh, Madhya Pradesh, Uttar Pradesh
East	Bihar, Jharkhand, Odisha, West Bengal
Northeast	Arunachal Pradesh, Assam, Manipur, Meghalaya, Mizoram, Nagaland, Sikkim, Tripura
West	Dadra and Nagar Haveli, Daman and Diu, Goa, Gujarat, Maharashtra
South	Andaman and Nicobar, Andhra Pradesh, Karnataka, Kerala, Lakshadweep, Puducherry, Tamil Nadu, Telangana

**Table A2:** Interpregnancy interval of sex of the children born in past five years, India, NFHS 2019-21, NFHS 2015-16, and NFHS 2005-06 (pooled data)

		Interpregnancy interval										
		**< 12 months**		**12–17 months**		**18–23 months**		**24–35 months**		**36–59 months**		**N**
		**Percent**	**N**	**Percent**	**N**	**Percent**	**N**	**Percent**	**N**	**Percent**	**N**	
Neonatal death sample	Male	34.4	31,329	25.5	23,862	16.8	15,937	17.1	16,337	6.2	6,135	**93,600**
	Female	34.4	28,600	25.3	21,939	16.7	14,849	17.2	15,168	6.4	5,791	**86,347**
Postneonatal death sample	Male	38.3	25,088	27.1	18,309	16.7	11,440	14.9	10,240	3.1	2,164	**67,241**
	Female	38.1	22,859	27.0	16,844	16.7	10,659	15.3	9,587	3.1	1,939	**61,888**
Diarrhea and/or ARI sample	Male	33.5	29,641	25.5	23,153	17.0	15,582	17.5	16,155	6.5	6,284	**90,815**
	Female	33.5	27,005	25.2	21,291	16.9	14,578	17.6	15,044	6.8	5,934	**83,852**
Stunting sample	Male	33.7	27,664	25.7	21,578	17.0	14,422	17.4	14,758	6.2	5,536	**83,958**
	Female	33.7	25,357	25.4	20,014	16.9	13,590	17.5	13,918	6.5	5,315	**78,194**
Underweight sample	Male	33.6	27,923	25.7	21,814	17.0	14,586	17.4	15,024	6.3	5,713	**85,060**
	Female	33.6	25,569	25.3	20,166	16.9	13,762	17.6	14,135	6.6	5,435	**79,067**

**Table A3:** Distribution of outcomes by interpregnancy interval stratified by sex of the child, India, NFHS 2019-21, NFHS 2015-16, and NFHS 2005-06 (pooled data)

	**Neonatal death**		**Postneonatal death**		**Diarrhea and/or ARI**		**Stunting**		**Underweight**	
	**Male**	**Female**	**Male**	**Female**	**Male**	**Female**	**Male**	**Female**	**Male**	**Female**
**Interpregnancy interval**										
< 12 months	4.1	3.6	1.8	2.3	13.6	12.4	43.2	41.9	37.8	37.5
12–17 months	2.6	2.3	1.3	1.5	13.2	12.9	43.8	43.0	39.6	38.1
18–23 months	2.5	1.7	1.2	1.5	14.0	12.9	40.9	38.7	38.2	35.5
24–35 months	2.2	1.9	1.3	1.2	13.8	12.9	37.6	35.2	35.5	32.9
36–59 months	2.7	2.1	1.9	2.7	14.3	13.1	31.1	23.9	30.1	24.5

**Table A4:** Logistic regression analysis of association of interpregnancy interval with infant mortality by sex of the child, India, NFHS 2019-21, NFHS 2015-16, and NFHS 2005-06 (pooled data)

	**Neonatal death**		**Postneonatal death**	
	**Male**	**Female**	**Male**	**Female**
**Binary logistic regression** ^**#**^				
**Interpregnancy interval**				
< 12 months	1.66*(1.43,1.92)	2.23*(1.87,2.65)	1.65*(1.28,2.13)	1.59*(1.27,2.00)
12–17 months	1.04(0.88,1.22)	1.36*(1.13,1.63)	1.14(0.85,1.54)	0.94(0.73,1.21)
18–23 months®	1.00	1.00	1.00	1.00
24–35 months	0.87(0.73,1.05)	1.10(0.89,1.35)	1.14(0.85,1.53)	0.77(0.58,1.02)
36–59 months	1.15(0.90,1.46)	1.28(0.96,1.70)	1.69*(1.12,2.55)	1.75*(1.14,2.68)

**Note**: - *p < 0.05; ® Reference

^**#**^Results are adjusted for Mother’s age at conception, Mother’s height, Mother’s schooling, Birth order, child wanted, Wealth quintiles, Religion, Caste, Place of residence, State-region, Survey round

**Table A5:** Logistic regression analysis of association of interpregnancy interval with child health outcomes by sex of child, India, NFHS 2019-21, NFHS 2015-16, and NFHS 2005-06 (pooled data)

**Covariate & category**	**Occurrence of diarrhea and/or acute respiratory infection**		**Stunting**		**Underweight**	
	Male	Female	Male	Female	Male	Female
**Binary logistic regression** ^**#**^						
**Interpregnancy interval**						
< 12 months	1.11* (1.03,1.20)	1.11* (1.02,1.21)	1.13* (1.06,1.20)	1.13* (1.06,1.20)	1.02 (0.96,1.09)	1.10* (1.03,1.17)
12–17 months	0.98 (0.90,1.06)	1.06 (0.97,1.15)	1.07* (1.01,1.13)	1.10* (1.03,1.17)	1.02 (0.96,1.08)	1.05 (0.98,1.11)
18–23 months®	1.00	1.00	1.00	1.00	1.00	1.00
24–35 months	0.95 (0.87,1.03)	0.95 (0.87,1.05)	1.02 (0.95,1.09)	1.01 (0.94,1.09)	0.97 (0.91,1.04)	0.98 (0.92,1.05)
36–59 months	0.99 (0.89,1.12)	0.98 (0.86,1.11)	1.11* (1.01,1.22)	0.90* (0.80,0.99)	0.95 (0.86,1.04)	0.85* (0.76,0.94)

**Note**: - *p < 0.05; ® Reference

^**#**^Results are adjusted for Mother’s age at conception, Mother’s height, Mother’s schooling, Birth order, child wanted, Wealth quintiles, Religion, Caste, Place of residence, State-region, Survey round

## Code Availability

STATA codes may be provided on request.
